# Supplementary material for: Nocturnal scent in a ‘bird-fig’: A cue to attract bats as additional dispersers?
Source: PLoS One. 2019 Aug 15;14(8):e0220461. doi: 10.1371/journal.pone.0220461 (PMC6695144; doi:10.1371/journal.pone.0220461)
Supplement: S1 File — Table A. Median and mean absolute deviation (mad) of relative amounts of 12 chemical compounds. Scent was collected from unripe fruits during night and from ripe fruits during night or day. Table B. Factor loadings of 12 volatile compounds on four principal components; factor loadings > 0.5 are shown. Table C. Standardized canonical discriminant function coefficients of four principal components. (PDF) [file pone.0220461.s001.pdf]

Table A. Median and mean absolute deviation (mad) of relative amounts of 12 chemical compounds. Scent was collected from unripe fruits during night and from ripe fruits during night or day

	unripe during night		ripe during night		ripe during day	
Substance	median	mad	median	mad	median	mad
1-dodecanol	4.7	2.71	4.01	4.11	2.4	1.6
1-tetradecanol	15.18	3.9	25.75	11.19	21	7.52
secondary alcohol	0	0	0	0	1.16	1.72
Nonanal	18.83	10.04	16.54	12.27	29.07	9.62
Decanal	6.14	4.67	4.84	2.16	9.17	4.94
unidentified substance	2.64	1.35	1.92	2.03	4.33	2.64
α-copaene	10.88	8.87	3.14	2.63	3.38	1.33
β-copaene + naphtalene derivative	7.41	4.7	7.32	5.09	3.46	2.83
α-cubebene + 1,1'-biphenyl	2.73	2.48	5.04	3.74	2.82	1.89
sesquiterpene A	6.84	4.42	10.56	9.06	4.74	4.51
β-selinene	2.4	1.33	1.13	0.65	0.71	0.58
δ-cadinene + calamenene	12.25	7.65	4.4	2.18	4.53	2.54

Table B. Factor loadings of 12 volatile compounds on four principal components; factor loadings > 0.5 are shown

	PC1	PC2	PC3	PC4
β-copaene + naphthalene derivative	0.913			
sesquiterpene A	0.912			
α-cubebene + 1,1'-biphenyl	0.857			
nonanal	-0.733			
decanal	-0.727			
α-copaene		0.825		
δ-cadinene + calamenene		0.789		
1-tetradecanol			-0.819	
1-dodecanol			-0.656	
secondary alcohol				0.836
unidentified substance				0.662
Cumulative proportion of variance	31.5	50.3	63.6	76.2

Table C. Standardized canonical discriminant function coefficients of four principal components

principal component	function	
	1	2
1	0.366	-0.737
2	0.948	0.293
3	0.059	0.663
4	-0.562	0.298
